# The Rényi Entropies Operate in Positive Semifields

**DOI:** 10.3390/e21080780

**Published:** 2019-08-08

**Authors:** Francisco J. Valverde-Albacete, Carmen Peláez-Moreno

**Affiliations:** Department of Signal Theory and Communications, Universidad Carlos III de Madrid, 28911 Leganés, Spain

**Keywords:** shifted Rényi entropy, Pap’s g-calculus, non-Newtonian calculus, positive commutative semifields, idempotent semifields, artificial intelligence, machine learning, computational intelligence

## Abstract

We set out to demonstrate that the Rényi entropies are better thought of as operating in a type of non-linear semiring called a positive semifield. We show how the Rényi’s postulates lead to Pap’s g-calculus where the functions carrying out the domain transformation are Rényi’s information function and its inverse. In its turn, Pap’s g-calculus under Rényi’s information function transforms the set of positive reals into a family of semirings where “standard” product has been transformed into sum and “standard” sum into a power-emphasized sum. Consequently, the transformed product has an inverse whence the structure is actually that of a positive semifield. Instances of this construction lead to idempotent analysis and tropical algebra as well as to less exotic structures. We conjecture that this is one of the reasons why tropical algebra procedures, like the Viterbi algorithm of dynamic programming, morphological processing, or neural networks are so successful in computational intelligence applications. But also, why there seem to exist so many computational intelligence procedures to deal with “information” at large.

## 1. Introduction

Some non-linear algebras like the min-plus semiring [1] have wide application in applications trying to model intelligent behaviour, among other domains [2]. In this paper we fuse ideas from the theory of the means, the abstract algebra of semirings and information theory to propose the following explanation for their ubiquity: *that they are in fact the natural algebras in which each of the infinite instances of the Rényi informations [3]:*

(1) $$H_{\alpha }\left(P_{X}\right)=\frac{1}{1-\alpha }\log\left(\sum_{i=1}^{n}p_{i}^{\alpha }\right)$$

*operate so that applications are “matched” to specific values of the order parameter α. From this starting point, the quasi-genetic, social process of scientific and technological advance selects which value of the parameter is most suited to make a technique work in a particular application.*

We will argue in this paper that these non-standard algebras are positive semifields, a special type of semiring with multiplicative inverses but no additive inverses. From the point of view of abstract algebra, recall that a semiring is an algebra S=〈S,⊕,⊗,ϵ,e〉 whose additive structure 〈S,⊕,ϵ〉 is a commutative monoid and whose multiplicative structure 〈S\{ϵ},⊗,e〉 is a monoid with multiplication distributing over addition from right and left and an additive neutral element absorbing for ⊗, i.e., ∀a∈S,ϵ⊗a=ϵ [4]. A semiring is commutative if its product is commutative. All semirings considered in this paper are commutative, whence we will drop the qualification altogether. A semiring is zero sum-free if whenever a sum is null all summands are also null, and entire if it has no non-null factors of zero. A semiring is positive if it is zero sum-free and entire [2]. Finally, a semiring is a semifield if there exists a multiplicative inverse for every element a∈S—notated as $a^{-1}$—except the null. All semifields are entire.

In this paper we concern ourselves with positive semifields, e.g., entire, zero sum-free semirings with a multiplicative inverse but no additive inverse (see Section 2.2), whose paragon is the set of non-negative reals with its standard operations $R_{\geq 0}=\langle \left[0,\infty \right),+,\times ,\cdot^{-1},0,1\rangle$. Their scientific and engineering interest lies in the fact that positive semifield applications abound in many areas of research. To cite but a few examples:

- Artificial intelligence (AI) [5] is an extensive field under which applications abound dealing with minimizing costs or maximizing utilities. Semifields and their dual-orderings (see Section 2.2 and Section 2.2.3) provide a perspective to mix these two kinds of valuations.
- Machine learning (ML) [6] makes heavy use of Probability Theory, which is built around the positive semifield of the non-negative reals with their standard algebra $R_{\geq 0}$ and negative logarithms thereof—called log-probabilities or log-likelihoods depending on the point of view—both of which are positive semifields, as shown in Section 2.2, Section 3.1.1, and Section 3.2.1.
- Computational intelligence (CI) [7] makes heavy use of positive semirings in the guise of fuzzy semirings. Although semifields cannot be considered “fuzzy” for several technical reasons, the name is sometimes an umbrella term under which non-standard algebras are included, many of which are semifields, e.g., the morphological semifield of morphological processing and memories, a special case of the semifields in Section 3.1.
- Other applications of positive semifields not related to modeling intelligence include Electrical Network analysis and synthesis (see the example in Section 2.2), queuing theory [8] and flow shop scheduling [1].

To build a basis for our initial conjecture, in methodological Section 2 we revisit a couple of seemingly unrelated observations: First, a simple shifting r=α−1 of the Rényi order parameter makes the connection between these entropies and the weighted Hölder means transparent [9] (Section 2.1). Second, the Rényi procedure to justify the form of the entropy [10] dovetails seamlessly into Pap’s g-calculus [11] as a construction on positive semifields (Section 2.2).

In the results Section 3 we conjoin both observations to support our hypothesis: we first briefly introduce an “entropic” semifield (Section 3.1.1) prior to proving, secondly, how the shifted Rényi entropy takes values in positive semifields, and also how this reflects back on the semifield of positive numbers interpreted as “probabilities” (Section 3.1.2). Third we use the newly defined semifield notation in two application instances: one, to provide an exception-free definition of the means for the extended positive numbers (Section 3.2.2), and two, to rewrite and provide a brief analysis into the Viterbi algorithm in terms of the semifield of max-information (Section 3.2.1). Finally we unfold the argumentation for our conjecture that AI, ML and CI applications are mostly dealing with Rényi entropies of different order (Section 3.3) and we trace some related art spanning the last 70 years (Section 3.4). We end the paper with a discussion of the issues touched upon it and some conclusions.

## 2. Materials and Methods

### 2.1. The Shifted Rényi Entropy

We first take the standpoint of information theory. Recall that the weighted power or Hölder mean of order *r* [12] is defined as

(2) $$\begin{array}{c} M_{r}\left(\vec{w},\vec{x}\right)={\left(\frac{\sum_{i=1}^{n}w_{i}\cdot x_{i}^{r}}{\sum_{k}w_{k}}\right)}^{\frac{1}{r}}={\left(\sum_{i=1}^{n}\frac{w_{i}}{\sum_{k}w_{k}}\cdot x_{i}^{r}\right)}^{\frac{1}{r}}. \end{array}$$

When r→0 the geometric mean appears:

$$M_{0}\left(\vec{w},\vec{x}\right)=\lim_{r\to 0}M_{r}\left(\vec{w},\vec{x}\right)={\left(\Pi_{i=1}^{n}x_{i}^{w_{i}}\right)}^{\frac{1}{\sum_{k}w_{k}}}.$$

Important cases of the means for historical and practical reasons and their relation to the (shifted and original) Rényi entropy are shown in Table 1.

To leverage the theory of generalized means to our advantage, we start with a correction to Rényi’s entropy definition: in [9] a case is made for shifting the original statement from the index that Rényi proposed α to r=α−1. Specifically, the transformation function for the average of surprisals [10] in the Rényi entropy (1) is arbitrary in the parameter α chosen for it and we may substitute r=α−1 to obtain the pair of formulas:

(3) $$\begin{array}{cc} \varphi^{\prime }\left(h\right)=b^{-rh}\; & {\varphi^{\prime }}^{-1}\left(p\right)=\frac{-1}{r}\log_{b}p. \end{array}$$

Note that the basis of the logarithm is usually not theoretically important. This is what we mean when we use logx or $\log_{b}x$ for an unspecified *b*.

Consider the extension of the surprisal, or Hartley’s function, to non-negative numbers as

(4) $$\begin{array}{cc} I_{*}\left(\cdot \right):[0,\infty ]\to [-\infty ,\infty ]\; & p\mapsto I_{*}\left(p\right)=-\log_{b}p\;. \end{array}$$

This is an antitone, one-to-one function from [0,∞] onto [−∞,∞], with inverse ${\left(I_{*}\right)}^{-1}\left(h\right)=b^{-h}$ for h∈[−∞,∞]. It is also the inverse of (3) for r=1. The following definitions are thus obtained:

**Definition** **1.** *Let $P_{X}\left(x_{i}\right)=p_{i}$ and $Q_{X}\left(y_{i}\right)=q_{i}$ be distributions with compatible support. Then the expression of the shifted Rényi entropy ${\tilde{H}}_{r}\left(P_{X}\right)$, cross-entropy ${\tilde{X}}_{r}\left(P_{X}∥Q_{X}\right)$, and divergence ${\tilde{D}}_{r}\left(P_{X}∥Q_{X}\right)$ are:*

(5) $$\begin{array}{ccc} {\tilde{H}}_{r}\left(P_{X}\right)=I_{*}\left(M_{r}\left(P_{X},P_{X}\right)\right)\; & {\tilde{X}}_{r}\left(P_{X}∥Q_{X}\right)=I_{*}\left(M_{r}\left(P_{X},Q_{X}\right)\right)\; & {\tilde{D}}_{r}\left(P_{X}∥Q_{X}\right)=-I_{*}\left(\frac{P_{X}}{Q_{X}}\right) \end{array}$$

Several brief points are worth mentioning in this respect, although the full argument can be followed in [9]:

- The properties of the Rényi entropy, therefore, stem from those of the mean, inversion and the logarithm.
- This is not merely a cosmetic change, since it has the potential to allow the simplification of issues and the discovery of new ones in dealing with the Rényi magnitudes. For instance, since the means are defined for all r∈[−∞,∞] there cannot be any objection to considering negative values for the index of the shifted entropy. This motivates calling ${\tilde{H}}_{r}\left(P_{X}\right)$ the Rényi spectrum (of entropy).
- The definition makes it also evident that the shifted cross-entropy seems to be the more general concept, given that the shifted entropy and divergence are clearly instances of it.
  $$\begin{array}{cc} {\tilde{H}}_{r}\left(P_{X}\right)={\tilde{X}}_{r}\left(P_{X}∥P_{X}\right)\; & {\tilde{D}}_{r}\left(P_{X}∥Q_{X}\right)=-{\tilde{X}}_{r}\left(P_{X}∥P_{X}/Q_{X}\right) \end{array}$$
- Also, the following lemma from [9] shows that the Rényi entropies can be rewritten in terms of the Shannon cross entropy and the Kullback–Leibler divergence:
  **Lemma** **1.** *Let r,s∈R∪{±∞}, $P_{X}\in \Delta^{n-1}$ where $\Delta^{n-1}$ is the simplex over the support $\mathrm{supp}\left(X\right)$ and ${\tilde{q}}_{r}\left(P_{X}\right)={\left\{\frac{p_{i}p_{i}^{r}}{\sum_{k}p_{k}p_{k}^{r}}\right\}}_{i=1}^{n}$ are the escort probabilities [9,13]. Then,*
  (6) $$\begin{array}{cc} {\tilde{H}}_{r}\left(P_{X}\right) & =\frac{1}{r}{\tilde{D}}_{0}\left({\tilde{q}}_{r}\left(P_{X}\right)∥P_{X}\right)+{\tilde{X}}_{0}\left({\tilde{q}}_{r}\left(P_{X}\right)∥P_{X}\right) \end{array}$$
  (7) $$\begin{array}{cc} {\tilde{H}}_{r}\left(P_{X}\right) & =\frac{-1}{r}{\tilde{H}}_{0}\left({\tilde{q}}_{r}\left(P_{X}\right)\right)+\frac{r+1}{r}{\tilde{X}}_{0}\left({\tilde{q}}_{r}\left(P_{X}\right)∥P_{X}\right). \end{array}$$
- Lemma (1) rewrites the entropies in terms of the geometric means which is, by no means, the only rewriting possible. Indeed, some would say that the arithmetic mean is more natural, and this is the program of information theoretic learning [14], where it is explored under the guise of $H_{2}\left(P_{X}\right)\equiv {\tilde{H}}_{1}\left(P_{X}\right)$.
- The shifting clarifies the relationship between quantities around the Rényi entropy: given (4), from every measure of information an equivalent average probability emerges. In particular:
  **Definition** **2.** *Let $X∼P_{X}$ with Rényi spectrum ${\tilde{H}}_{r}\left(P_{X}\right)$. Then the equivalent probability function of ${\tilde{P}}_{r}\left(P_{X}\right)$ is the Hartley inverse of ${\tilde{H}}_{r}\left(P_{X}\right)$ over all values of r∈[−∞,∞]*
  (8) $$\begin{array}{c} {\tilde{P}}_{r}\left(P_{X}\right)={\left(I_{*}\right)}^{-1}\left({\tilde{H}}_{r}\left(P_{X}\right)\right)=M_{r}\left(P_{X},P_{X}\right). \end{array}$$
- Similarly, the information potential ${\tilde{V}}_{r}\left(P_{X}\right)=E_{P_{X}}\left\{P_{X}^{r}\right\}$ has been independently motivated in a number of applications [14]. The next lemma is immediate using the conversion function (3) on the shifted entropy (5).
  **Lemma** **2.** *Let $X∼P_{X}$. The information potential is the $\varphi^{\prime }$ image of the shifted Rényi entropy*
  (9) $$\begin{array}{c} {\tilde{V}}_{r}\left(P_{X}\right)=E_{P_{X}}\left\{P_{X}^{r}\right\}=\sum_{i}\frac{p_{i}}{\sum_{k}p_{k}}p_{i}^{r}=b^{-r{\tilde{H}}_{r}\left(P_{X}\right)}=\varphi^{\prime }\left({\tilde{H}}_{r}\left(P_{X}\right)\right). \end{array}$$

**Figure 1:**
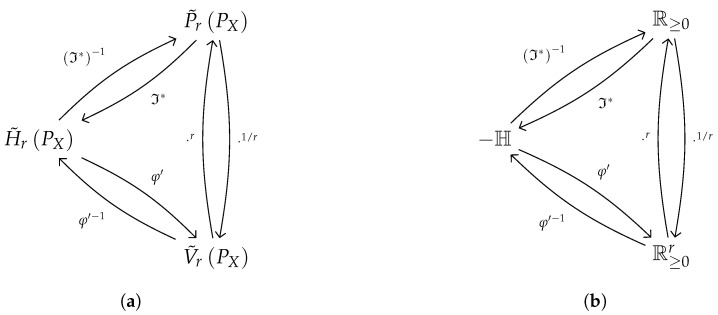
Schematics of relationship due to entropic isomorphisms. (**a**) Between shifted entropy-related quantities (from [9]). (**b**) Between entropy-related domains (see Section 3.1).

These three quantities—the shifted Rényi entropy, the equivalent probability function, and the information potential—stand in a relationship, as described in Figure 1a, whose characterization is the conducting thread of this paper. In [9] other formulas to convert them into each other are tabulated.

### 2.2. Positive Semifields

We now retake the point of view of abstract algebra. From the material in Section 2.1 it seems evident that non-negative quantities are important for our purposes. Non-negativity is captured by the concept of zero sum-free semiring mentioned above, but we focus in the slightly less general notion of dioid (for double monoid) [2] where there is an order available that interacts “nicely” with the operations of the algebra.

#### 2.2.1. Complete and Positive Dioids

A dioid is a commutative semiring D where the canonical preorder relation—a≼b if and only if there exists c∈D with a⊕c=b—is actually an order 〈D,≼〉. In a dioid the canonical orden relation is compatible with both ⊕ and ⊗ ([2], Chapter 1, Prop. 6.1.7) and the additive zero epsilon is always the infimum of the dioid or bottom hence the notation ϵ=infD=⊥. Dioids are all zero sum-free, that is, they have no non-null additive factors of zero: if a,b∈D,a⊕b=ϵ then a=ϵ and b=ϵ.

A dioid is complete if it is complete as an ordered set for the canonical order relation, and the following distributivity properties hold, for all A⊆D,b∈D,

(10) $$\begin{array}{cc} \left(\underset{a\in A}{⨁}a\right)⊗b=\underset{a\in A}{⨁}\left(a⊗b\right)\; & b⊗\left(\underset{a\in A}{⨁}a\right)=\underset{a\in A}{⨁}\left(b⊗a\right) \end{array}$$

In complete dioids, there is already a top element $⊤={⊕}_{a\in D}a$.

A semiring is entire or zero-divisor free if a⊗b=ϵ implies a=ϵ or b=ϵ. If the dioid is entire, its order properties justifies calling it a positive dioid or information algebra [2].

#### 2.2.2. Positive Semifields

A semifield, as mentioned in the introduction, is a semiring whose multiplicative structure $\langle K\backslash \left\{\epsilon \right\},⊗,e,\cdot^{⊛}\rangle$ is a group, where $\cdot^{⊛}:K\to K$ is the function to calculate the inverse such that $\forall u\in K,u⊗u^{⊛}=e$. Since all semifields are entire, dioids that are at the same time semifields are called positive semifields, of which the positive reals or rationals are a paragon.

**Example** **1** (Semifield of non-negative reals)**.** *The nonnegative reals $R_{\geq 0}=\langle \left[0,\infty \right),+,\times ,\cdot^{⊛},⊥=0,e=1\rangle$ are the basis for the computations in Probability Theory and other quantities that are multiplicatively aggregated. The 0 has no inverse hence $R_{\geq 0}$ is incomplete. Consider modeling utilities and costs with this algebra. For utilities 0 acts as a least element—a bottom—and the order is somewhat directed “away” from this element hence the underlying order is 〈[0,∞),≤〉, that is to say utilities are to be maximized. But its dual order 〈[0,∞),≥〉—intended to model costs, to be minimized—has no bottom (see below), hence applications using, for instance, multiplicative costs and utilities at the same time, will be difficult to carry out in this algebra and notation.*

**Figure 2:**
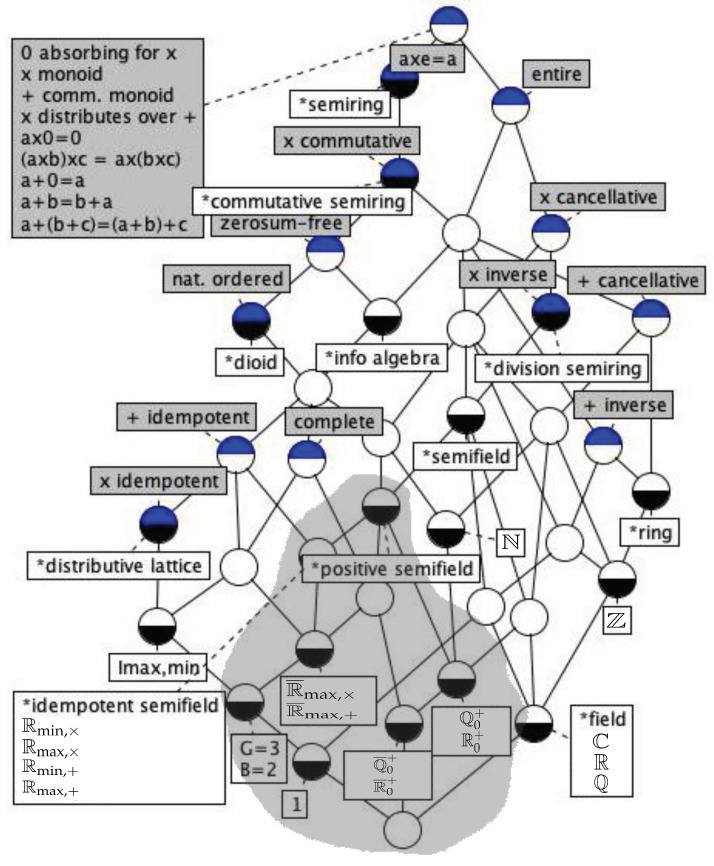
Lattice of a selection of abstract (leading asterisk, white label) and concrete (white label) commutative semirings and their properties (grey label) mentioned in the text. Adapted from [15]. Each node is a concept of abstract algebra: its properties are obtained from the gray labels in nodes upwards, and its structures from the white labels in nodes downwards. The picture is related to the chosen sets of properties and algebras and does not fully reflect the structure of the class of semirings. We have chosen to highlight positive semifields, like $R_{0}^{+}$ and $Q_{0}^{+}$.

Figure 2 shows a concept lattice of the position of positive semifields within the commutative semirings, as well as the better known collateral families of fields like R and distributive lattices like 〈[0,1],max,min〉 (cfr. ([15], Figure 1)).

In incomplete semifields like the one above, the inverse of the bottom element is the “elephant in the room” to be avoided in computations. Fortunately, semiring theory provides a construction to supply this missing element in semifields [4]. However, the problem with the dual order in the semifield mentioned above suggests that we introduce both completions at the same time through the following theorem (the need for the dotted notation will be made clear after the theorem).

**Theorem** **1.** *For every incomplete positive semifield $K=\langle K,⊕,⊗,\cdot^{⊛},⊥,e\rangle$*

*1.*  *There is a pair of completed semifields over $\bar{K}=K\cup \left\{⊤\right\}$*
  (11) $$\begin{array}{cc} \bar{K}=\langle K,\underset{\cdot }{⊕},\underset{\cdot }{⊗},\cdot^{⊛},⊥,e,⊤\rangle \; & {\bar{K}}^{⊛}=\langle K,\overset{\cdot }{⊕},\overset{\cdot }{⊗},\cdot^{⊛},⊤,e,⊥\rangle \end{array}$$
  *where $⊤={⊥}^{⊛}$ and $⊥={⊤}^{⊛}$ by definition,*
*2.*  *In addition to the individual laws as positive semifields, we have the modular laws:*
  (12) $$\begin{array}{cc} \left(u\underset{\cdot }{⊕}v\right)\underset{\cdot }{⊗}\left(u\overset{\cdot }{⊕}v\right)=u\underset{\cdot }{⊗}v\; & \left(u\underset{\cdot }{⊕}v\right)\overset{\cdot }{⊗}\left(u\overset{\cdot }{⊕}v\right)=u\overset{\cdot }{⊗}v \end{array}$$
  *the analogues of the De Morgan laws:*
  (13) $$\begin{array}{cc} u\underset{\cdot }{⊕}v={\left(u^{⊛}\overset{\cdot }{⊕}v^{⊛}\right)}^{⊛}\; & u\overset{\cdot }{⊕}v={\left(u^{⊛}\underset{\cdot }{⊕}v^{⊛}\right)}^{⊛}\\ u\underset{\cdot }{⊗}v={\left(u^{⊛}\overset{\cdot }{⊗}v^{⊛}\right)}^{⊛}\; & u\overset{\cdot }{⊗}v={\left(u^{⊛}\underset{\cdot }{⊗}v^{⊛}\right)}^{⊛} \end{array}$$
  *and the self-dual inequality in the natural order*
  (14) $$u\underset{\cdot }{⊗}\left(v\overset{\cdot }{⊗}w\right)≼\left(u\underset{\cdot }{⊗}v\right)\overset{\cdot }{⊗}w\;.$$
*3.*  *Further, if K is a positive dioid, then the inversion operation is a dual order isomorphism between the dual order structures $\bar{K}=\langle K,≼\rangle$ and ${\left(\bar{K}\right)}^{⊛}=\langle K,≽\equiv {≼}^{\delta }\rangle$ with the natural order of the original semifield a suborder of the first structure.*

**Proof.**  For 1, consider $\bar{K}=K\cup \left\{⊤\right\}$ obtained by adding a top element to *K*. The order $\langle \bar{K},≼\rangle$ is extended with x≼⊤,∀x∈K, hence the notation ⊤ for this element. In this completed set the definition of the operations are (in this paper, cases in a definition-by-case should be interpreted from top to bottom: this first case to match applies):

$$\begin{array}{cccc} u\underset{\cdot }{⊕}v & =\left\{\begin{array}{cc} ⊤ & u=⊤\;\mathrm{or}\;v=⊤\\ u⊕v & u,v\in K\backslash \{⊤\} \end{array}\right.\; & u\underset{\cdot }{⊗}v & =\left\{\begin{array}{cc} ⊥ & u=⊥\;\mathrm{or}\;v=⊥\\ ⊤ & u=⊤\;\mathrm{or}\;v=⊤\\ u⊗v & u,v\in K\backslash \{⊥,⊤\}\; \end{array}\right. \end{array}$$

with the inversion operation completed by the definition of $⊤={⊥}^{⊛}$ and $⊥={⊤}^{⊛}$. So $\bar{K}=\langle K,\underset{\cdot }{⊕},\underset{\cdot }{⊗},\cdot^{⊛},⊥,e,⊤\rangle$ is the well-known top-completion of a positive semiring ([4], p. 250).

In this construction, inversion is total, injective and surjective in the completed domain $\bar{K}$, hence a bijection. It is easily seen as an involution ${\left(x^{⊛}\right)}^{⊛}=x,\forall x\in \bar{K}$, in fact, the inverse for the order-dual ${\bar{K}}^{⊛}$. But, the operations in the inverse semifield are given by:

(15) $$\begin{array}{cc} a\overset{\cdot }{⊕}b={\left(a^{⊛}\underset{\cdot }{⊕}b^{⊛}\right)}^{⊛}\; & a\overset{\cdot }{⊗}b={\left(a^{⊛}\underset{\cdot }{⊗}b^{⊛}\right)}^{⊛}. \end{array}$$

To gain an understanding of the operations, we explore their results on a case-by-case basis. For $\overset{\cdot }{⊕}$:

- If a=⊤ then $a^{⊛}\underset{\cdot }{⊕}b^{⊛}=⊥\underset{\cdot }{⊕}b^{⊛}=b^{⊛}$, whence $⊤\overset{\cdot }{⊕}b=b$, and symmetrically for b=⊤. That is, ⊤ is the neutral element of addition $\overset{\cdot }{⊕}$ and $\langle \bar{K},\overset{\cdot }{⊕},⊤\rangle$ a monoid.
- If a=⊥, then ${⊥}^{⊛}\underset{\cdot }{⊕}b^{⊛}=⊤\underset{\cdot }{⊕}b^{⊛}=⊤$, whence $⊥\overset{\cdot }{⊕}b={⊤}^{⊛}=⊥$ and symmetrically for b=⊥. This proves that ⊥ is the maximum element of ${≼}^{\delta }$, to be defined below.
- Otherwise, for {a,b}⊆K\{⊥}, we have $a\overset{\cdot }{⊕}b=\frac{1}{a^{⊛}⊕b^{⊛}}=\frac{a⊗b}{a⊕b}$,

while for $\overset{\cdot }{⊗}$:

- If a=⊤ then $a^{⊛}\underset{\cdot }{⊗}b^{⊛}=⊥\underset{\cdot }{⊗}b^{⊛}=⊥$, whence $⊤\overset{\cdot }{⊗}b={⊥}^{⊛}=⊤$, and symmetrically for *b*.
- If a=⊥ but b≠⊤, then $a^{⊛}\underset{\cdot }{⊗}b^{⊛}=⊤\underset{\cdot }{⊗}b^{⊛}=⊤$, whence $a\overset{\cdot }{⊗}b=⊥$.
- Otherwise, for {a,b}⊆K\{⊥}, we have $a\overset{\cdot }{⊗}b=a⊗b=a\underset{\cdot }{⊗}b$.

Note that $\langle \bar{K},\overset{\cdot }{⊕},⊤\rangle$ is a commutative monoid, with commutativity and associativity following from those of $\underset{\cdot }{⊕}$. Likewise, $e^{⊛}=e$ is easily proven to be the neutral element of $\langle \bar{K},\overset{\cdot }{⊗},e,\cdot^{⊛}\rangle$ which is a commutative group with commutativity and associativity issuing from those of $\underset{\cdot }{⊗}$, whose inverse is the involution $\cdot^{⊛}$. So to prove that the algebra is a semifield we only need to prove the distributive of $\overset{\cdot }{⊗}$ over $\overset{\cdot }{⊕}$, for $u,v,z\in \bar{K}$:

$$\begin{array}{cc} u\overset{\cdot }{⊗}\left(v\overset{\cdot }{⊕}z\right) & =\frac{1}{u^{⊛}\underset{\cdot }{⊗}{\left({\left(v^{⊛}\underset{\cdot }{⊕}z^{⊛}\right)}^{⊛}\right)}^{⊛}}=\frac{1}{u^{⊛}\underset{\cdot }{⊗}\left(v^{⊛}\underset{\cdot }{⊕}z^{⊛}\right)}=\frac{1}{u^{⊛}\underset{\cdot }{⊗}v^{⊛}\underset{\cdot }{⊕}u^{⊛}\underset{\cdot }{⊗}z^{⊛}}\\ & ={\left(u^{⊛}\underset{\cdot }{⊗}v^{⊛}\right)}^{⊛}\overset{\cdot }{⊕}{\left(u^{⊛}\underset{\cdot }{⊗}z^{⊛}\right)}^{⊛}=u\overset{\cdot }{⊗}v\overset{\cdot }{⊕}u\overset{\cdot }{⊗}z. \end{array}$$

Therefore ${\bar{K}}^{⊛}=\langle K,\overset{\cdot }{⊕},\overset{\cdot }{⊗},\cdot^{⊛},⊤,e,⊥\rangle$ is another completed semifield issuing from the first one.

For 2, the proof for the De Morgan-like laws is easy from the definion of $\overset{\cdot }{⊕}$ and $\overset{\cdot }{⊗}$: those definitions are actually one half of the laws, e.g., $a\overset{\cdot }{⊗}b={\left(a^{⊛}\underset{\cdot }{⊗}b^{⊛}\right)}^{⊛}$. Inverting and by the involutivity of the inversion $a^{⊛}\underset{\cdot }{⊗}b^{⊛}={\left(a\overset{\cdot }{⊗}b\right)}^{⊛}$, we change $a=u^{⊛}$ and $b=v^{⊛}$ to prove the result. The proof is analogue for the multiplicative law. The dual equalities (12) and the self-dual inequality (14) are just exercises in case analysis.

For 3 we want to find the order for the dual semifield, ${≼}^{\delta }=≽$.

- if {u,v}⊆K\{⊥}, since the natural order is compatible with multiplication we multiply by $u^{⊛}\underset{\cdot }{⊗}v^{⊛}$ to obtain $u⊗\left(u^{⊛}⊗v^{⊛}\right)≼v⊗\left(u^{⊛}⊗v^{⊛}\right)$ whence, by cancellation, $v^{⊛}≼u^{⊛}$, or else $u^{⊛}{≼}^{\delta }v^{⊛}$, so the order is the dual on inverses.
- We have that $⊥≼v,\forall v\in \bar{K}$, otherwise $v{≼}^{\delta }⊥$ which asserts that $⊥={⊤}^{⊛}$ is the “top” of the inverted order. Likewise we read from $u≼⊤,\forall u\in \bar{K}$ that $⊤{≼}^{\delta }u$, that is $⊤={⊥}^{⊛}$ is the “bottom” in ${≼}^{\delta }$.

whence ${\langle \bar{K},≼\rangle }^{⊛}=\langle \bar{K},{≼}^{\delta }\rangle$. □

This proof provides extensive guide on how to use the notation. Note that:

- The dot notation, from [16], is a mnemonic for where do the multiplication of the bottom and top go:
  $$\begin{array}{cc} ⊥\underset{\cdot }{⊗}⊤=⊥\; & ⊥\overset{\cdot }{⊗}⊤=⊤ \end{array}$$
  implying that the “lower” addition and multiplication are aligned with the original order in the semifield, e.g., $⊥\underset{\cdot }{⊗}x=⊥$ while the “upper” addition and multiplication are aligned with its dual. All other cases remain as defined for ⊕ in the incomplete semifield.
- The case analysis for the operators in the dual semifield allows us to write their definition-by-cases as follows:
  (16) $$\begin{array}{cc} a\overset{\cdot }{⊕}b=\left\{\begin{array}{cc} b & a=⊤\\ a & b=⊤\\ ⊥ & a=⊥\;\mathrm{or}\;b=⊥\\ \frac{1}{a^{⊛}⊕b^{⊛}} & \{a,b\}\subseteq K\backslash \{⊥\}\; \end{array}\right.\; & a\overset{\cdot }{⊗}b=\left\{\begin{array}{cc} ⊤ & a=⊤\;\mathrm{or}\;b=⊤\\ ⊥ & a=⊥\;\mathrm{or}\;b=⊥\\ a⊗b & \{a,b\}\subseteq K\backslash \{⊥\}\; \end{array}\right.. \end{array}$$
  This is important for calculations, but notice that $\overset{\cdot }{⊗}$ and $\underset{\cdot }{⊗}$ only differ in the corner cases.
- The notation to “speak” about these semirings tries to follow a convention reminiscent of that of boolean algebra, where the inversion is the complement ([17], Chapter 12).
- Note that $\underset{\cdot }{⊕}$ and $\overset{\cdot }{⊕}$ seem to operate on different “polarities” of the underlying set: if one operates on two numbers, the other operates on their inverses while this is not so for the respective multiplications. This proves extremely important to model physical quantities and other concepts with these calculi (see example below).

Regarding the intrinsic usefulness of completed positive semifields that are not fields—apart from the very obvious but degenerate case of B, the booleans—we have the following example used, for instance, in convex analysis and electrical network theory.

**Example** **2** (Dual semifields for the non-negative reals)**.** *The previous procedure shows that there are some problems with the notation of Example 1, and this led to the definition of the following signatures for this semifield and its inverse in convex analysis [16]:*

(17) $$\begin{array}{cc} R_{\geq 0}=\langle \left[0,\infty \right],\underset{\cdot }{+},\underset{\cdot }{\times },\cdot^{-1},0,1,\infty \rangle \; & R_{\geq 0}^{-1}=\langle \left[0,\infty \right],\overset{\cdot }{+},\overset{\cdot }{\times },\cdot^{-1},\infty ,1,0\rangle . \end{array}$$

*Both of these algebras are used, for instance, in electrical engineering (EE), the algebra of complete positive reals to carry out the series summation of resistances, and its dual semifield to carry out parallel summation of conductances. With the convention that $R_{\geq 0}$ semiring models resistances, it is easy to see that the bottom element, ⊥=0 models a shortcircuit, that the top element ⊤=∞ models an open circuit (infinite resistance) and these conventions are swapped in the dually-ordered semifield of conductances. Since EE does not use the dotted notation explained in this paper, the formulas required for the multiplication of the extremes:*

$$\begin{array}{cc} 0\underset{\cdot }{\times }\infty =0\; & 0\overset{\cdot }{\times }\infty =\infty , \end{array}$$

*are not allowed in circuit analysis. In our opinion, this strongly suggests that what is actually being operated with are the incomplete versions of these semifields, and the many problems that EE students have in learning how to properly deal with these values may stem from this fact. Other uses in Economics are detailed in [17].*

**Example** **3** (Multiplicatively and additively idempotent costs and utilities)**.** *Several pairs of such order-dual semirings are known, for instance:*

- *The completed max-times and min-times semifields.*
  (18) $$\begin{array}{cc} {\bar{R}}_{\max,\times }=\langle \left[0,\infty \right],\max,\underset{\cdot }{\times },\cdot^{-1},0,1,\infty \rangle \; & {\bar{R}}_{\min,\times }=\langle \left[0,\infty \right],\min,\overset{\cdot }{\times },\cdot^{-1},\infty ,1,0\rangle . \end{array}$$
- *The completed max-plus (schedule algebra, polar algebra) and min-plus semifields (tropical algebra).*
  (19) $$\begin{array}{cc} {\bar{R}}_{\max,+}=\langle \left[-\infty ,\infty \right],\max,\underset{\cdot }{+},-\cdot ,-\infty ,0,\infty \rangle \; & {\bar{R}}_{\min,+}=\langle \left[-\infty ,\infty \right],\min,\overset{\cdot }{+},-\cdot ,\infty ,0,-\infty \rangle . \end{array}$$

*Note that their additions are all idempotent: a semiring with idempotent addition is simply called an idempotent semiring and it is always positive. These find usage in path-finding algorithms, and some more examples can be found in [2]. The mechanism whereby they are exposed as pairs of dually ordered semifields is explained in Section 2.2.3. For an example of their use in a particular application, see Section 3.2.1.*

#### 2.2.3. A Construction for Positive Semifields

There is a non-countable number of semifields obtainable from $R_{\geq 0}$. Their discovery is probably due to Maslov and collaborators ([18], §1.1.1), but we present here the generalized procedure introduced by Pap and others [11,19,20], but see also Section 3.4.

**Construction** **1** (Pap’s dioids and semifields)**.** *Let $R_{\geq 0}$ be the semiring of non-negative reals, and consider a strictly monotone generator function g on an interval [a,b]⊆[−∞,∞] with endpoints in [0,∞]. Since g is strictly monotone it admits an inverse $g^{-1}$, so set*

*1.*  *the pseudo-addition, $u⊕v=g^{-1}(g\left(u\right)\underset{\cdot }{+}\left(g\left(v\right)\right)$*
*2.*  *the pseudo-multiplication, $u⊗v=g^{-1}(g\left(u\right)\underset{\cdot }{\times }\left(g\left(v\right)\right)$*
*3.*  *neutral element, $e=g^{-1}\left(1\right)$*
*4.*  *inverse, $x^{⊛}=g^{-1}\left(\frac{1}{g(x)}\right)$,*

*then,*

*1.*  *if g is strictly monotone and increasing increasing such that g(a)=0 and g(b)=∞, then a complete positive semifield whose order is aligned with that of $R_{\geq 0}$ is:*
  $${\bar{K}}_{g}=\langle \left[a,b\right],\underset{\cdot }{⊕},\underset{\cdot }{⊗},\cdot^{⊛},⊥=a,e,⊤=b\rangle \;.$$
*2.*  *order-dually, if g is strictly monotone and decreasing such that g(a)=∞ and g(b)=0, then a complete positive semifield whose order is aligned with that of $R_{\geq 0}^{-1}$ is*
  $${\bar{K}}_{g}^{⊛}=\langle \left[a,b\right],\overset{\cdot }{⊕},\overset{\cdot }{⊗},\cdot^{⊛},{⊥}^{⊛}=b,e,{⊤}^{⊛}=a\rangle \;.$$

**Proof.**  See [11,19] for the basic dioid ${\bar{K}}_{g}$, and ([2], p. 49) for the inverse operation and the fact that it is a semifield, hence a positive semifield. The description of the operations of ${\bar{K}}_{g}^{⊛}$ is provided by Theorem 1. □

Note how in the *g*-calculus operations are always named with a “pseudo-” prefix, but it does not agree with Semiring Theory practice, hence we drop it. Also, the effect of the type of motonicity of *g* is to impose the polarity of the extended operations. Remember that the inversion $\cdot^{⊛}$ is a dual isomorphism of semifields, so that ${\left({\bar{K}}_{g}^{⊛}\right)}^{⊛}={\bar{K}}_{g}$ and ${\left({\bar{K}}_{g}\right)}^{⊛}={\bar{K}}_{g}^{⊛}$. The different notation for the underlying inverse $\cdot^{-1}$ and the inverse in Pap’s construction $\cdot^{⊛}$ is introduced so that it can later be instantiated in a number of constructed inverses, as follows.

Our use of Construction 1 is to generate different kind of semifields by providing different generator functions:

**Construction** **2** (Multiplicative-product real semifields [20])**.** *Consider a free parameter r∈[−∞,0)⋃(0,∞] and the function $g\left(x\right)=x^{r}$ in [a,b]=[0,∞] in Construction 1. For the operations we obtain:*

(20) $$\begin{array}{ccc} u{⊕}_{r}v={\left(u^{r}\underset{\cdot }{+}v^{r}\right)}^{\frac{1}{r}}\; & u{⊗}_{r}v={\left(u^{r}\underset{\cdot }{\times }v^{r}\right)}^{\frac{1}{r}}=u\underset{\cdot }{\times }v\; & u^{⊛}={\left(\frac{1}{u^{r}}\right)}^{\frac{1}{r}}=u^{-1}, \end{array}$$

*where the basic operations are to be interpreted in $R_{\geq 0}$. Now,*

- *if r∈(0,∞] then $g\left(x\right)=x^{r}$ is strictly monotone increasing whence ${⊥}_{r}=0$, $e_{r}=1$, and ${⊤}_{r}=\infty$, and the complete positive semifield generated, order-aligned with $R_{\geq 0}$, is:*
  (21) $$\begin{array}{c} {\left(R_{\geq 0}\right)}_{r}=\langle \left[0,\infty \right],{\underset{\cdot }{⊕}}_{r},\underset{\cdot }{\times },\cdot^{-1},{⊥}_{r}=0,e,{⊤}_{r}=\infty \rangle . \end{array}$$
- *if r∈[−∞,0) then $g\left(x\right)=x^{r}$ is strictly monotone decreasing whence ${⊥}_{r}=\infty$, $e_{r}=1$, and ${⊤}_{r}=0$, and the complete positive semifield generated, order-aligned with ${\left(R_{\geq 0}\right)}^{-1}$, or dually aligned with $R_{\geq 0}$, is:*
  (22) $$\begin{array}{cc} {\left(R_{\geq 0}\right)}_{-r} & ={{\left(R_{\geq 0}\right)}_{r}}^{-1}={\left(R_{\geq 0}^{-1}\right)}_{r}=\langle \left[0,\infty \right],{\overset{\cdot }{⊕}}_{r},\overset{\cdot }{\times },\cdot^{-1},{⊥}_{r}^{-1}=\infty ,e,{⊤}_{r}^{-1}=0\rangle . \end{array}$$

**Proof.**  By instantiation of the basic case. See the details in [21]. □

Note that ${\left(R_{\geq 0}\right)}_{1}\equiv R_{\geq 0}$ and ${\left(R_{\geq 0}\right)}_{-1}\equiv R_{\geq 0}^{-1}$. This suggests the following corollary:

**Corollary** **1.** *${\left(R_{\geq 0}\right)}_{r}$ and ${\left(R_{\geq 0}\right)}_{-r}$ are inverse, completed positive semifields.*

In particular, consider the cases:

**Corollary** **2.** *In the previous Construction 2,*

(23) $$\begin{array}{cc} {\left(R_{\geq 0}\right)}_{1}=R_{\geq 0}\; & {\left(R_{\geq 0}\right)}_{-1}=R_{\geq 0}^{-1} \end{array}$$

(24) $$\begin{array}{cc} \lim_{r\to \infty }{\left(R_{\geq 0}\right)}_{r}={\bar{R}}_{\max,\times }\; & \lim_{r\to -\infty }{{\left(R_{\geq 0}\right)}_{r}}^{-1}={\bar{R}}_{\min,\times } \end{array}$$

**Proof.**  The proof of (23) by inspection. For (24) see [20]. □

All these semifields have the same product, and the same “extreme” points, {0,1,∞}. Their only difference lies in the addition. Sometimes, when only the product is important in an application, the addition remains in the background and we are not really sure in which algebra we are working on. Note also that instead of using the abstract notation for the inversion $\cdot^{⊛}$, since $R_{\geq 0}$ is the paragon originating all other behaviour, we have decided to use the original notation for the inversion in the (incomplete) semifield.

The case where r=0 deserves to be commented. To start with, note that for q>0, ${\left(R_{\geq 0}\right)}_{q}$ is aligned with $R_{\geq 0}$ while ${\left(R_{\geq 0}\right)}_{-q}$ is aligned with ${\left(R_{\geq 0}\right)}^{\delta }$, therefore $\lim_{r\to 0^{+}}{\left(R_{\geq 0}\right)}_{r}$ and $\lim_{r\to 0^{-}}{\left(R_{\geq 0}\right)}_{r}$ are endowed with opposite orderings. Therefore the following lemma is not a surprise.

**Lemma** **3.** *${\left(R_{\geq 0}\right)}_{0}$ is not a semifield.*

**Proof.**  Recall, that on finite operands $u{⊕}_{r}v={\left(u^{r}+v^{r}\right)}^{1/r}$. Then we may generalize, due to the associativity of the operation to a vector $\vec{v}={\left\{v_{i}\right\}}_{i}$ to ${⨁}_{r,i}v_{i}={\left(\sum_{i}v_{i}^{r}\right)}^{1/r}$. Next, consider $\vec{w},\vec{x}\in {\left(0,\infty \right)}^{n}$, and q∈(0,∞). From the properties of the means (see also [12], C. 2), it is easy to prove that

$$\sqrt[{-q}]{\sum_{i=1}^{n}w_{i}x_{i}^{-q}}\leq \prod_{i=1}^{n}x_{i}^{w_{i}}\leq \sqrt[{q}]{\sum_{i=1}^{n}w_{i}x_{i}^{q}}.$$

By considering $w_{i}=1$ and introducing the limits as q→0 we have

(25) $$\begin{array}{c} \underset{0,i}{⨁}v_{i}=\prod_{i}v_{i}, \end{array}$$

whence the additive and the multiplicative structures of ${\left(R_{\geq 0}\right)}_{0}$ are the same, so the structure is not even a semiring. Furthermore, when u,v∈[0,∞], then $\lim_{r\to 0^{-}}u{⊕}_{r}v=u\overset{\cdot }{\times }v$ whereas $\lim_{r\to 0^{+}}u{⊕}_{r}v=u\underset{\cdot }{\times }v$. Hence, in particular when, say u=0 and v=∞ we have $\infty =u\overset{\cdot }{⊗}v≮u\underset{\cdot }{⊗}v=0$ and the product is clearly not defined. □

## 3. Results

We are now ready to start presenting a chain of results that leads to our conjecture.

### 3.1. Entropic Semifields

#### 3.1.1. The Basic Entropic Semifield

The effect of Hartley’s information function is to induce from the set of positive numbers (restricted to the [0,1] interval) a semifield of the extended reals [0,∞]. To see this, we actually consider it acting on the whole of the non-negative reals $R_{\geq 0}$ of (17) onto the algebra of entropies denoted by H.

**Theorem** **2** (Hartley’s semifields)**.** *The algebra $\langle \left[-\infty ,\infty \right],⊕,⊗,\cdot^{⊛},\infty ,0\rangle$ with*

(26) $$h_{1}⊕h_{2}=h_{1}+h_{2}-\ln\left(e^{h_{1}}+e^{h_{2}}\right)\;h_{1}⊗h_{2}=h_{1}+h_{2}\;h^{⊛}=-h,$$

*obtained from that of positive numbers by Hartley’s information function is a positive semifield that can be completed in two different ways to two mutually dual semifields:*

(27) $$H=\langle \left[-\infty ,\infty \right],\underset{\cdot }{⊕},\underset{\cdot }{⊗},-\cdot ,⊥=-\infty ,e=0,⊤=\infty \rangle -H=\langle \left[-\infty ,\infty \right],\overset{\cdot }{⊕},\overset{\cdot }{⊗},-\cdot ,-⊥=\infty ,e=0,-⊤=-\infty \rangle$$

*whose elements can be considered as entropic values and operated accordingly.*

**Proof.**  Recall the extension of Hartley’s information function to non-negative numbers in (4), with logarithm base b=e. Since ${\left(I_{*}\right)}^{-1}\left(h\right)=e^{-h}$ is monotone, it is a generator (function) for Construction 1, with the following addition, multiplication and inversion:

$$\begin{array}{cc} h_{1}⊕h_{2} & =I_{*}\left({\left(I_{*}\right)}^{-1}\left(h_{1}\right)+{\left(I_{*}\right)}^{-1}\left(h_{2}\right)\right)=-\ln\left(e^{-h_{1}}+e^{-h_{2}}\right)=\ln\frac{e^{h_{1}+h_{2}}}{e^{h_{1}}+e^{h_{2}}}=h_{1}+h_{2}-\ln\left(e^{h_{1}}+e^{h_{2}}\right)\\ h_{1}⊗h_{2} & =I_{*}\left({\left(I_{*}\right)}^{-1}\left(h_{1}\right)\times {\left(I_{*}\right)}^{-1}\left(h_{2}\right)\right)=-\ln\left(e^{-h_{1}}\cdot e^{-h_{2}}\right)=-\ln\left(e^{-(h_{1}+h_{2})}\right)=h_{1}+h_{2}\\ h^{⊛} & =I_{*}\left(\frac{1}{{\left(I_{*}\right)}^{-1}\left(h\right)}\right)=-\ln\frac{1}{e^{-h}}=-h\;. \end{array}$$

The “interesting” points {0,e,∞} are transformed as:

(28) $$\begin{array}{ccc} I_{*}\left(0\right)=\infty \; & I_{*}\left(1\right)=0\; & I_{*}\left(\infty \right)=-\infty \end{array}$$

The rest follows by Construction 1 and Theorem 1. □

Several considerations are worth stating. First, we have not restricted this dual-order isomorphism to the sub-semiring of probabilities on purpose. Despite this, on $I_{*}\left([0,1]\right)=\left[0,\infty \right]\subseteq \left[-\infty ,\infty \right]$ our intuitions about amounts of informations hold; we still believe that the “information” in $I^{*}\left(0\right)=\infty$ is the highest, whereas the probability of ${\left(I^{*}\right)}^{-1}\left(\infty \right)=0$ is the smallest.

Second, the notation for the inverse of Hartley’s semifield −H is a mnemonic to remind that this is a semifield in which the inversion is actually an additive inverse, and consequently the product is an addition.

Third, the order of $R_{\geq 0}$ is aligned with that of H, actually a sub-order of it in the abstract algebra sense: same properties on a subset of the original carrier set. But the order of −H is aligned with the dual order, that of $R_{\geq 0}^{-1}$. This has to be taken into consideration in the application of Theorem 1 to the proof of Theorem 2. Corollary 3 makes this difference clear.

**Corollary** **3.** *Hartley’s information function is a dual-order isomorphism of completed positive semifields.*

**Proof.**  In the proof of the previous theorem, note that $I_{*}\left(\cdot \right)$ is monotonically decreasing, entailing that the construction inverts orders in semifields, e.g., $I_{*}\left(R_{\geq 0}\right)=-H$ and $I_{*}\left(R_{\geq 0}^{-1}\right)=H$. □

**Figure 3:**
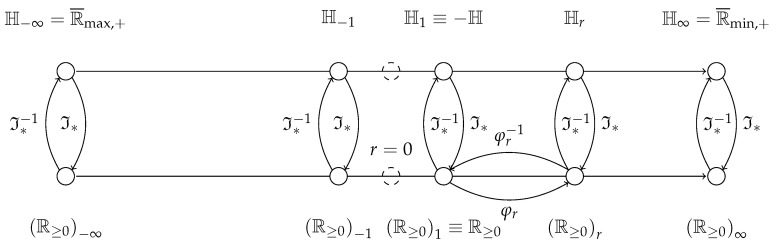
Domain diagram to interpret the Rényi transformation in the context of the expectations of probabilities and informations. The horizontal axes represent the evolution of parameter *r*. Notice that for r=0 no positive semifields are found (dashed circles). Above lie the entropic semirings, below, the semirings that are deformations of $R_{\geq 0}$. Between each of these pairs with the same *r*—where only a few pairs for r∈{−∞,−1,1,r,∞} have been made explicit—mediate Hartley’s information function $I_{*}\left(\cdot \right)$ and its inverse ${\left(I_{*}\right)}^{-1}\left(\cdot \right)$. In the semifields below, only the case for the deformation between $R_{\geq 0}$ and ${\left(R_{\geq 0}\right)}_{r}$ is shown in full.

Figure 3 provides a graphic depiction of the relationship of the domains described by Theorem 2 and Corollary 3, as well as the entropy domains described by Theorem 3 below. It also provides a more comprehensive picture than Figure 1b).

#### 3.1.2. Constructed Entropic Semifields

The next important fact is that Rényi’s modified averaging function $\varphi \left(p\right)=\frac{-1}{r}\log_{b}p$ and its inverse are also dual isomorphisms of positive semifields. To prove this we make said functions appear in the construction of the semifields as generators.

**Theorem** **3** (Additive-product real semifields or Entropy semifields)**.** *Let r∈[−∞,∞]\{0} and b∈(1,∞). Then the algebra $\langle \left[-\infty ,\infty \right],{⊕}_{r},{⊗}_{r},\cdot^{-1},⊥=\infty ,e=0\rangle$ whose basic operations are:*

(29) $$\begin{array}{ccc} u{⊕}_{r}v=u+v-\log_{b}{\left(b^{ru}+b^{rv}\right)}^{\frac{1}{r}}\; & u{⊗}_{r}v=u+v\; & u^{⊛}=-u \end{array}$$

*can be completed to two dually-ordered positive semifields*

(30) $$\begin{array}{cc} H_{r} & =\langle \left[-\infty ,\infty \right],{\underset{\cdot }{⊕}}_{r},{\underset{\cdot }{⊗}}_{r},-\cdot ,⊥=-\infty ,e=0,⊤=\infty \rangle \end{array}$$

(31) $$\begin{array}{cc} -H_{r} & =\langle \left[-\infty ,\infty \right],{\overset{\cdot }{⊕}}_{r},{\overset{\cdot }{⊗}}_{r},-\cdot ,-⊥=\infty ,e=0,-⊤=-\infty \rangle \end{array}$$

*whose elements can be considered as emphasized, entropic values and operated accordingly.*

**Proof.**  We build these semifields with a composition of results. The first one is the well known result from the theory of functional means that we choose to cast into the framework of Pap’s *g*-calculus: the power mean of order *r* is the pseudo arithmetic-mean with generator $\varphi_{r}\left(x\right)=x^{r}$ and inverse $\varphi_{r}^{-1}\left(y\right)=y^{1/r}$. This was used in Construction 2 to build the semifields of (21) and (22). The second result is Corollary 3, where $I_{*}\left(\cdot \right)$ is proven a dual order isomorphism of semirings.

We next use the composition of functions $\varphi_{r}^{\prime }=I_{*}\circ \varphi_{r}$ and its inverse ${\left(\varphi_{r}^{\prime }\right)}^{-1}=\varphi_{r}^{-1}\circ I_{*}^{-1}$. The latter exists, since it is a composition of isomorphisms and it is a dual order isomorphism, since $I_{*}\left(\cdot \right)$ is order-inverting while $\varphi_{r}$ is not. That composition is precisely Rényi’s function $\varphi_{r}^{\prime }\left(h\right)=b^{-rh}$ with inverse ${\left(\varphi_{r}^{\prime }\right)}^{-1}\left(p\right)=\frac{-1}{r}\log_{b}p$, whence:

$$\begin{array}{cc} u{⊕}_{r}v & ={\left(\varphi_{r}^{\prime }\right)}^{-1}\left(\varphi_{r}^{\prime }\left(u\right)+\varphi_{r}^{\prime }\left(v\right)\right)=\frac{-1}{r}\log_{b}\left(b^{-ru}+b^{-rv}\right)=\frac{-1}{r}\log_{b}\left(\frac{b^{ru}+b^{rv}}{b^{r(u+v)}}\right)=u+v-\log_{b}{\left(b^{ru}+b^{rv}\right)}^{\frac{1}{r}}\\ u{⊗}_{r}v & ={\left(\varphi_{r}^{\prime }\right)}^{-1}\left(\varphi_{r}^{\prime }\left(u\right)\times \varphi_{r}^{\prime }\left(v\right)\right)=\frac{-1}{r}\log_{b}\left(b^{-ru}\times b^{-rv}\right)=\frac{-1}{r}\log_{b}\left(b^{-r(u+v)}\right)=u+v\\ u^{⊛} & ={\left(\varphi_{r}^{\prime }\right)}^{-1}\left(\frac{1}{\varphi_{r}^{\prime }\left(u\right)}\right)=\frac{-1}{r}\log_{b}\left(\frac{1}{b^{-ru}}\right)=-u \end{array}$$

This composition is strictly increasing when r∈[−∞,0) and strictly decreasing when r∈(0,∞], hence, when applying Construction 1 with it:

- for r∈(0,∞] we get $-H_{r}=I_{*}\left({\left(R_{\geq 0}\right)}_{r}\right)=\langle \left[-\infty ,\infty \right],{\overset{\cdot }{⊕}}_{r},{\overset{\cdot }{⊗}}_{r},-\cdot ,-⊥=\infty ,e=0,-⊤=-\infty \rangle$, and
- for r∈[−∞,0) we obtain $H_{r}=I_{*}\left({{\left(R_{\geq 0}\right)}_{r}}^{-1}\right)=\langle \left[-\infty ,\infty \right],{\underset{\cdot }{⊕}}_{r},{\underset{\cdot }{⊗}}_{r},-\cdot ,⊥=-\infty ,e=0,⊤=\infty \rangle$,

with the extended operations:

$$\begin{array}{cccc} -⊥ & =-(-\infty )=\infty =⊤ & -⊤ & =-\infty =⊥\\ u{\underset{\cdot }{⊕}}_{r}v & =\left\{\begin{array}{cc} \infty & \mathrm{if}\;u=\infty \;\mathrm{or}\;v=\infty \\ u{⊕}_{r}v & \mathrm{otherwise} \end{array}\right. & u{\underset{\cdot }{⊗}}_{r}v & =\left\{\begin{array}{cc} -\infty & \mathrm{if}\;u=-\infty \;\mathrm{or}\;v=-\infty \\ u+v & \mathrm{otherwise} \end{array}\right.\\ u{\overset{\cdot }{⊕}}_{r}v & =\left\{\begin{array}{cc} -\infty & \mathrm{if}\;u=-\infty \;\mathrm{or}\;v=-\infty \\ u{⊕}_{r}v & \mathrm{otherwise} \end{array}\right.\; & u{\overset{\cdot }{⊗}}_{r}v & =\left\{\begin{array}{cc} \infty & \mathrm{if}\;u=\infty \;\mathrm{or}\;v=\infty \\ u+v & \mathrm{otherwise} \end{array}\right. \end{array}$$

 □

Note that in these semirings we have: $-\infty {\underset{\cdot }{⊗}}_{r}\infty =-\infty$ and $-\infty {\overset{\cdot }{⊗}}_{r}\infty =\infty$ for r∈[−∞,∞]\{0}.

Moreau’s original proposals [16] are found for r=1 $-\infty \underset{\cdot }{\times }\infty =-\infty$ and $-\infty \overset{\cdot }{\times }\infty =\infty$. Further details for compositions of generating functions and other averaging constructions can be found in [22].

Similar to Corollary 2 we have the following:

**Proposition** **1.** *In the semifield construction of Theorem 3*

(32) $$\begin{array}{cc} H_{-1}=H\; & H_{1}=-H \end{array}$$

(33) $$\begin{array}{cc} \lim_{r\to -\infty }H_{r}={\bar{R}}_{\max,+}\; & \lim_{r\to \infty }H_{r}={\bar{R}}_{\min,+} \end{array}$$

**Proof.**  The proof of (32) is done by inspection. For (33), well-known, see [2,20]. □

The apparent incongruity of $H_{1}=-H$ stems from the different origins of each notation: $H_{1}$ comes from the theory of semirings and is due to the fact that we have forced it to be aligned with −H which is ultimately motivated by Shannon’s choice of sign for the entropy function [23]. Note that in thermodynamics, this choice of sign is the inverse, that is, negentropy is the privileged concept.

The following corollary is the important result we announced at the beginning of this section.

**Corollary** **4.** *The Rényi entropies in the Rényi spectrum ${\tilde{H}}_{r}\left(P_{X}\right)$ take values in the semifields $H_{r}$.*

**Proof.**  We notice that the generating function to use Construction 1 in Theorem 3 is none other than the function to calculate the Rényi non-linear average $\varphi^{\prime }$ of (3). Hence the values resulting from Rényi’s entropies belong in that semifield of Theorem 3 with the respective *r* parameter. □

Therefore Theorem 3 and Corollary 4 state that we have to use semifield algebra when carrying out calculations with entropies, otherwise said, they jointly confer meaning to the statement entropies operate on positive semifields in the title of this paper. An example follows in Section 3.2.1.

### 3.2. Applications

We would like to clarify further how entropies operate in positive semifields, and, in general, the importance of positive semifields for entropy. For this purpose we present two different applications of the theory of entropic semifields: one shows how to write entropy-processing algorithms in linear (semifield) algebra and the second shows why we cannot avoid positive semifields in entropy definitions, if we want to consider extreme values.

#### 3.2.1. Rewriting the Viterbi Algorithm in Semifields

In this application note we analyze rewriting the Viterbi algorithm [24] in semifield notation, following recent work presented in [25].

Introduction Initially devised as a teaching aid for convolutional codes [24], the “Viterbi” was soon proven to be an optimal algorithm for shortest-path decoding in a specific type of network [26]. When this network comes from the unfolding over time of a Markov chain, it can also be used to recover an “optimal” sequence of states and transition over a generative model for a given sequence of observations [27]. In this natural generalization, it has been applied to text and speech recognition and synthesis, among other cognitively-relevant applications that used to be considered part of “classical” AI but are modernly better tackled with more specialized ML or CI techniques.

Development. Consider a weighted finite automaton of *n* states over a finite alphabet Σ, $\left(\vec{p},W,\vec{b}\right)$ defined by

- A starting distribution $\vec{p}$, where ${\vec{p}}_{i},1\leq i\leq n$, is the probability of starting at state *i*.
- A transition matrix *W*, where $w_{ij}={\left(W\right)}_{ij},1\leq i,j\leq n$ is the probability of a transition from state *i* to state *j*, and
- An emission distribution $\vec{b}$, where $\vec{b}{\left(\alpha \right)}_{i}$ is the probability of emitting symbol α∈Σ from state i,1≤i≤n.

It is also possible to assign non-null probabilities for definite accepting states, but these can be assimilated to the more general framework used here.

Let $\sigma \in \Sigma^{+}$ be a non-empty string of length *T* from the free monoid built over Σ that we call the sequence of observations and call $\sigma_{t}$ the *t*-th observation. Supposing the sequence of observations $[\sigma_{t}],1\leq t\leq T$ to have been generated by the automaton, the purpose of the Viterbi algorithm is to find the best path score $\vec{q}\left(T\right)$ over the set of possible generation paths.

This may be done using Bellman’s Principle of Optimality [28] leading to the following recursion for 0≤t≤T

(34) $$\begin{array}{cc} {\vec{q}}_{i}\left(0\right) & ={\vec{p}}_{i}\\ {\vec{q}}_{i}\left(t\right) & =\max_{j}\left(w_{ij}\cdot {\vec{q}}_{j}\left(t-1\right)\right)\cdot {\vec{b}}_{i}\left(\sigma_{t}\right) \end{array}$$

It is easy to see that this equation can be written in ${\left(R_{\geq 0}\right)}_{\infty }\equiv {\bar{R}}_{\max,\times }$ as introduced in (18). First, for any vector $\vec{p}$, let $\Lambda (\vec{p})$ be a square matrix whose main diagonal is built with the components of $\vec{p}$, and whose off-diagonal entries are zero. Then we may write (34) in matrix form as

(35) $$\begin{array}{c} \vec{q}\left(t\right)=\Lambda \left(\vec{b}\left(\sigma_{t}\right)\right)\underset{\cdot }{\times }W^{t}\underset{\cdot }{\times }\vec{q}\left(t-1\right) \end{array}$$

where we have co-opted the scalar lower multiplication to represent also matrix multiplication, as customary in the theory of linear algebra over semirings [4].

To actually solve (35), Hartley’s function is customarily invoked on all the probabilities appearing in (34) (cfr. [25])—often citing the advantages of working on log-probabilities for numeric computing—to obtain

(36) $$\begin{array}{c} -\log{\vec{q}}_{i}\left(t\right)=\min_{j}\left(-\log w_{ij}\overset{\cdot }{+}\left(-\log{\vec{q}}_{j}\left(t-1\right)\right)\right)\overset{\cdot }{+}\left(-\log{\vec{b}}_{i}\left(\sigma_{t}\right)\right). \end{array}$$

Two remarks are pertinent here. First, a realization already put forward by many researchers [2]: this expression describes a non-linear computation—a minimum score in this case—by means of a linear equation in ${\bar{R}}_{\min,+}$ (19). Second, one of the main ideas put forward in this paper: due to the fact that the original equations dealt with probabilities and the nature of Hartley’s function, (37) is a linear equation on $H_{\infty }$ entropies.

Obviously, $H_{\infty }\equiv {\bar{R}}_{\min,+}$, hence, call $\vec{x}\left(t\right)=-\log\vec{q}\left(t\right)$, $H_{b}\left(\sigma \right)=-\log\Lambda \left(b\left(\sigma \right)\right)$ and $H_{W}=-\log W$, so that (36) can be rewritten in matrix form as

(37) $$\begin{array}{c} \vec{x}\left(t\right)=H_{b}\left(\sigma_{t}\right)\overset{\cdot }{⊗}{H_{W}}^{t}\overset{\cdot }{⊗}\vec{x}\left(t-1\right)\;. \end{array}$$

It is also illustrative to see the inclusion of a pruning threshold η in this computation by ${\bar{R}}_{\min,+}$-linear means [25]. By definition ${\vec{x}\left(t\right)}^{t}\overset{\cdot }{⊗}\vec{x}\left(t\right)=2\cdot \min_{i}x_{i}\left(t\right)$ so consider the vector ${\vec{\eta }}_{i}=\eta +\frac{1}{2}{\vec{x}\left(t\right)}^{t}\overset{\cdot }{⊗}\vec{x}\left(t\right),1\leq i\leq n$ and $X\left(t\right)=\Lambda (\vec{x}\left(t\right))$. The problem of thresholding the path computations can be stated as finding $\vec{y}$ such that $X\left(t\right)\overset{\cdot }{⊗}\vec{y}\geq \vec{\eta }$. The solution to this set of equations is well-known (see e.g., [1], Theorem 3.2.3 of on p. 58): for generic and conformant matrix *A* and vector $\vec{b}$ we have

$$\begin{array}{c} A\overset{\cdot }{⊗}\vec{y}\geq \vec{b}\Leftrightarrow \vec{y}\geq A^{⊛}\underset{\cdot }{⊗}\vec{b}\;. \end{array}$$

Note that for a matrix *A* with ${\bar{R}}_{\min,+}$ entries, the Cuninghame-Green conjugate $A^{⊛}=-A^{t}$ is the analogue of the complex conjugate [29], and that the maximal solution takes an analytical form in the dual semifield, in this case $H_{-\infty }\equiv {\bar{R}}_{\max,+}$.

Then the negative elements of $\vec{y}=X{\left(t\right)}^{⊛}\underset{\cdot }{⊗}\vec{\eta }$ indicate which components need to be pruned. That is, if the individual equations read ${\vec{y}}_{i}=-x_{i}\left(t\right)+\eta +\min_{j}x_{j}\left(t\right)$, setting in $x_{j}\left(t\right)=-\infty$ at step *t* for those indices of $\vec{y}$ which are negative actually carries out the pruning.

Discussion What we want to highlight with this application are several facts:

- Actual non-linear computations in the Viterbi algorithm take the form of linear operations over a particular semifield, used here to minimize costs in ${\bar{R}}_{\min,+}$. This takes the form of a linear matrix equation, an instance of “linear processing” in a non-linear algebra.
- Secondly and more importantly, because of Theorem 3 we know that the values in which the log-probabilities are being operated in the Viterbi are actually the Rényi entropies with index r=−∞. Hence we conclude that the Viterbi is a quantitative, information entropy-processing algorithm.
- Even such a non-linear process as pruning using a threshold can be characterized and carried out by linear processing in the semifield. This is an instance of the fact that linear operations in $H_{\infty }$ process information in (“standard” algebraic) non-linear ways.

Already the authors of [25] provide a geometric construction to characterize the search space associated to the Viterbi as described by ${\bar{R}}_{\min,+}$ equations in terms of half-spaces and their tropical polytopes. Our own work has proven that the spectral spaces for matrices like $H_{b}\left(\sigma_{t}\right)$ and $H_{W}$ are actually complete lattices instead of the better known orthogonal subspaces of the Singular Value Decomposition of matrices in “standard” algebra [15,30]. In fact, the left and right spectral spaces issued form the ${\bar{R}}_{\min,+}$ Singular Value Decomposition show a relation akin to that of extents and intents in Formal Concept Analysis [31]. Further exploration of these issues is left for future work.

Finally, a crucial issue is to realize that the “optimality” of the decoding strategy is prescribed by the algebra being used in the decoding—in the language of this paper, Viterbi is ${\bar{R}}_{\min,+}$- or $H_{\infty }$-optimal—and this is often based in a-priori considerations on the problem. It follows that several other algorithms can be built with the template of the Viterbi by changing the underlying semifield, but all require that the addition be idempotent, that is ∀a∈S,a⊕a=a ([2], Chapters 1 and 8).

#### 3.2.2. Rewriting the Hölder Means and Rényi Entropies with Semifields

Corollary 4 and Definition 1 suggest that the mean $M_{r}\left(\vec{w},\vec{x}\right)$ in (2) actually takes values in the positive semifield ${\left(R_{\geq 0}\right)}_{r}$. The surprisal function would then transform these values into the entropy semifield $H_{r}$. However, the expression in Definition 1 does not easily lend itself to accept the extreme values ∞ and 0. Therefore, we propose the following definition for the means in positive semifields:

**Definition** **3.** *Let $\vec{x},\vec{w}\in {\left[0,\infty \right]}^{n}$ and r∈[−∞,∞]. Then in the dual completed semifields issuing from $R_{\geq 0}$,*

(38) $$\begin{array}{cc} {\hat{M}}_{r}\left(\vec{w},\vec{x}\right) & =\left\{\begin{array}{cc} {\left({\sum^{•}}_{i}\left(w_{i}\overset{\;\;\;\cdot }{/}{\sum^{•}}_{k}w_{k}\right)\overset{\cdot }{⊗}x_{i}^{r}\right)}^{\frac{1}{r}} & r<0\\ e^{{\hat{M}}_{1}\left(\vec{w},\ln\vec{x}\right)} & r=0\\ {\left(\sum_{•i}\left(w_{i}\underset{\;\;\;\cdot }{/}\sum_{•k}w_{k}\right)\underset{\cdot }{⊗}x_{i}^{r}\right)}^{\frac{1}{r}} & r>0 \end{array}\right., \end{array}$$

*where the case for r=0 is only valid when there are no i≠j with $x_{i}^{w_{i}}=0$ and $x_{j}^{w_{j}}=\infty$.*

**Justification.** To justify this definition, we need to translate the painstakingly developed description of the means in ([12], Chapter II) into complete positive semifield notation to obtain the rewritten means ${\hat{M}}_{r}\left(\vec{w},\vec{x}\right)$. For that purpose, we will carry out a case-based analysis based in the following set definitions: let *n* be the dimension of $\vec{x}$ and $\vec{w}$ and $\bar{n}=\left\{1,\ldots ,n\right\}$ the full set of indices on all the components, and let:

(39) $$\begin{array}{ccc} B_{\vec{x}}=\{i\in \bar{n}|x_{i}=0\}\; & T_{\vec{x}}=\{i\in \bar{n}|x_{i}=\infty \}\; & F_{\vec{x}}=\{i\in \bar{n}|x_{i}\in \left(0,\infty \right)\} \end{array}$$

be, respectively, the set of indices of zero, finite, and infinite components, and notice that $\bar{n}=B_{\vec{x}}\cup F_{\vec{x}}\cup T_{\vec{x}}$.

Note that the corner cases when $\vec{x}=0$ are catered to by the reflexivity of the means [12].

In particular, when r>0, if $\vec{x}=0$, then $B_{\vec{x}}=\bar{n}$ and we require that ${\hat{M}}_{r}\left(\vec{w},\vec{x}\right)=0$. This entails that the multiplication in (38) for r>0 must be the lower multiplication so that

$${\hat{M}}_{r}\left(\vec{w},\vec{x}\right)={\left(\underset{i}{\sum_{•}}{\tilde{q}}_{0}\left(\vec{w}\right)\underset{\cdot }{⊗}0\right)}^{\frac{1}{r}}=0.$$

In [12], Chapter II, the existence of $w_{i}=0$ is directly disallowed, and, we take the same is the case for $w_{i}=\infty$. However, the authors later take into consideration the second possibility, so we have decided to include both from the start. For this purpose, consider the case where $B_{\vec{w}}=\bar{n}$. Let us first define $W=\sum_{•i}w_{i}$, which is the only possible definition consistent with our intuitions of an overall weight *W* for r>0, and notice that $B_{\vec{w}}=\bar{n}\Leftrightarrow W=0\Leftrightarrow W^{⊛}=\infty$. This means we could, in principle, define the normalized weight distribution ${\tilde{q}}_{0}\left(\vec{w}\right)$ in two possible ways:

1. either ${\tilde{q}}_{0}\left(\vec{w}\right)=\left\{w_{i}\underset{\;\;\;\cdot }{/}W\right\}=\left\{w_{i}\overset{\cdot }{⊗}W^{⊛}\right\}$;
2. or ${\tilde{q}}_{0}\left(\vec{w}\right)=\left\{w_{i}\overset{\;\;\;\cdot }{/}W\right\}=\left\{w_{i}\underset{\cdot }{⊗}W^{⊛}\right\}$.

In the first case, if all the weights are null, that is $B_{\vec{W}}=\bar{n}$ then $\forall i,w_{i}\overset{\cdot }{⊗}W^{⊛}=0\overset{\cdot }{⊗}\infty =\infty$. For zero coordinates of $\vec{x}$ this poses no problem, but for $j\in \bar{B_{\vec{x}}}\neq ⌀$ we find that $\left(w_{j}\overset{\cdot }{⊗}W^{⊛}\right)\underset{\cdot }{⊗}x_{j}^{r}=\infty \underset{\cdot }{⊗}x_{i}^{r}=\infty$ whence the whole summation is infinite. This does not seem reasonable. On the other hand, in the second case, we have that $\left(w_{i}\underset{\cdot }{⊗}W^{⊛}\right)\underset{\cdot }{⊗}x_{i}^{r}=\left(0\underset{\cdot }{⊗}\infty \right)\underset{\cdot }{⊗}x_{i}^{r}=0\underset{\cdot }{⊗}x_{i}^{r}=0$, as expected.

However, this has too much “annihilating power” for if $T_{\vec{w}}\neq ⌀\Leftrightarrow W=\infty \Leftrightarrow W^{⊛}=0$, considering an $i\in T_{\vec{w}}$ such that $w_{i}=\infty$, then every factor is erased since for $j\in \bar{B_{\vec{w}}}$ is $\left(w_{j}\underset{\cdot }{⊗}W^{⊛}\right)\underset{\cdot }{⊗}x_{j}^{r}=\left(w_{j}\underset{\cdot }{⊗}0\right)\underset{\cdot }{⊗}x_{j}^{r}=0$, and the alternative is even less intuitive than the preceding one. Therefore we are forced to choose alternative 1 ${\tilde{q}}_{0}\left(\vec{w}\right)=\left\{w_{i}\underset{\;\;\;\cdot }{/}W\right\}$ with the caveat that when all of the weights are null (a strange situation indeed) this does not make sense.

Two points are worth stating:

- The case where r<0 is reasoned out by duality, with $T_{\vec{x}}$ being dual to $B_{\vec{x}}$, r<0 being the dual to r>0, ∞ dual to 0, and the upper multiplication and addition duals to the lower multiplication and addition. In the following we just use this “duality” argument to solve the case for r<0.
- The rest of the cases to analyze essentially have $B_{\vec{x}}\neq \bar{n}$ and $B_{\vec{w}}\neq \bar{n}$ whence their complements are non-null $\bar{B_{\vec{x}}}\neq ⌀,\bar{B_{\vec{w}}}\neq ⌀$. This means that the actual summation in (38) is extended to $\bar{B_{\vec{x}}}\cap \bar{B_{\vec{w}}}$, with $\bar{B_{\vec{x}}}=F_{\vec{x}}\cup T_{\vec{x}}$ and $\bar{B_{\vec{w}}}=F_{\vec{w}}\cup T_{\vec{w}}$.

Due to Theorem 1 and Construction 1 we know that if $F_{\vec{x}}=\bar{B_{\vec{x}}}$ and $F_{\vec{w}}=\bar{B_{\vec{w}}}$—that is, all non-zero coordinates are finite—then the expressions reduce to those of the classical definition in (2) so that ${\hat{M}}_{r}\left(\vec{w},\vec{x}\right)=M_{r}\left(\vec{w},\vec{x}\right)$ for r∈[−∞,∞], that is including r≤0.

**Figure 4:**
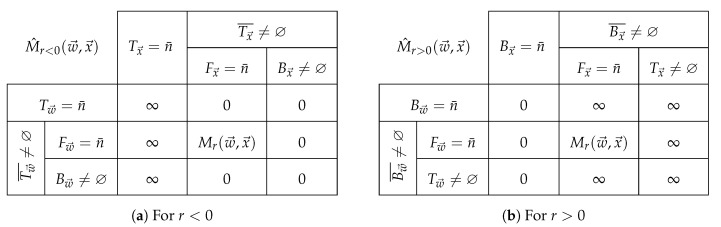
A summary of cases for ${\hat{M}}_{r}\left(\vec{w},\vec{x}\right)$ (**a**) for r<0 and (**b**) for r>0

The only cases left to analyze for r>0 are those where ∞ appears in either the weights $\vec{w}$ or the quantities $\vec{x}$ on subindices $i\in \bar{B_{\vec{x}}}\cap \bar{B_{\vec{w}}}$. Notice that if $i\in T_{\vec{x}}$ then $\left(w_{i}\overset{\cdot }{⊗}W^{⊛}\right)\underset{\cdot }{⊗}\infty =\infty$ since $w_{i}\overset{\cdot }{⊗}W^{⊛}$ cannot be zero, therefore ${\hat{M}}_{r}\left(\vec{w},\vec{x}\right)=\infty$. Likewise, if $i\in T_{\vec{w}}$ then $W=\infty \Leftrightarrow W^{⊛}=0$ and $w_{i}\overset{\cdot }{⊗}W^{⊛}=\infty \overset{\cdot }{⊗}0=\infty$ whence the factor $\left(w_{i}\overset{\cdot }{⊗}W^{⊛}\right)\underset{\cdot }{⊗}x_{i}^{r}=\infty \underset{\cdot }{⊗}x_{i}^{r}=\infty$ and ${\hat{M}}_{r}\left(\vec{w},\vec{x}\right)=\infty$. We have collected all cases for r>0, in Figure 4, along with those for r<0 obtained by duality.

Only the case r=0 is left for analysis, and recall this is $M_{0}\left(\vec{w},\vec{x}\right)={\left(\Pi_{i}x_{i}^{w_{i}}\right)}^{\frac{1}{\sum_{k}w_{k}}}$. In [12] this case is treated exceptionally throughout the treatise, and one of the often used alternative expressions for it is $M_{0}\left(\vec{w},\vec{x}\right)=\exp\left\{\sum_{i}\frac{w_{i}}{W}\ln x_{i}\right\}$ leading immediately to $M_{0}\left(\vec{w},\vec{x}\right)=\exp\left\{M_{1}\left(\vec{w},\ln\vec{x}\right)\right\}$ where the logaritm has to be interpreted entry-wise.

Recalling that log(·) and exp(·) are order isomorphisms of semifields, this suggests that we are trying to solve the hard problem for r=0 in the semifield H by using r=1 thanks to the properties of the logarithm. However, the addition in H is a multiplication so the rules for calculating the weighted means there might be different, considering that $\log x_{i}$ might be negative, or, specially, that factors like $w_{i}\cdot \log x_{i}$ are actually H exponentiations, i.e., they come from factors $\log(x_{i}^{w_{i}})$.

In fact, the clue to suggest the form for this mean comes from information theory and the requirement that $0\cdot \log\frac{1}{0}=0$, necessary to ignore impossible events with $p_{i}=0$ with $-p_{i}\cdot \log p_{i}=0$ in the entropy calculation. This can be modeled in our framework by demanding that this multiplication be $0\underset{\cdot }{⊗}\log\left(1\underset{\;\;\;\cdot }{/}0\right)=0\underset{\cdot }{⊗}\infty =0$ and to write the geometric mean as:

(40) $$\begin{array}{cc} {\hat{M}}_{0}\left(\vec{w},\vec{x}\right) & =\exp\{{\hat{M}}_{1}\left(\vec{w},\ln\vec{x}\right)\} \end{array}$$

Note that since the logarithm changes the base semifield for ${\hat{M}}_{1}\left(\vec{w},\log\vec{x}\right)$ the Figure 4b has to be reinterpreted, along with the sets of subindices: if the components of a vector $\vec{x}$ belong to a semifield K with carrier set *K*, then the index sets have to be defined with respect to the semifield in it. Since the carrier set of H is $R_{\pm \infty }=\left[-\infty ,\infty \right]$ then they read as:

$$\begin{array}{ccc} B_{\vec{x}}=\{i\in \bar{n}|x_{i}={⊥}_{R_{\pm \infty }}=-\infty \}\; & T_{\vec{x}}=\{i\in \bar{n}|x_{i}={⊤}_{R_{\pm \infty }}=\infty \}\; & F_{\vec{x}}=\{i\in \bar{n}|x_{i}\in \left({⊥}_{R_{\pm \infty }},{⊤}_{R_{\pm \infty }}\right)\} \end{array}$$

So a table similar to Figure 4b for ${\hat{M}}_{1}\left(\vec{w},\log\vec{x}\right)$ would have −∞ instead of 0. Nevertheless, the exponentiation would bring the mean back to $R_{\geq 0}$, and entailing that (40) actually follows Figure 4b.

This concludes our justification of the casting of the weighted means into semifield algebra. Note that a single $\vec{x}$ may have $x_{i}=0$ and $x_{j}=\infty$ for i≠j, a case explicitly addressed in [12]: ${\hat{M}}_{r}\left(\vec{p},\vec{x}\right)=\infty \underset{\cdot }{⊗}u\left(r\right)$ where u(r) is the step function, undefined at r=0

$$u\left(r\right)=\left\{\begin{array}{cc} 0 & r<0\\ 1 & r>0 \end{array}\right..$$

With this formulation we are now capable of describing the Rényi entropies in semifield notation:

**Definition** **4.** *Let $P_{X}\left(x_{i}\right)=p_{i}$ and $Q_{X}\left(y_{i}\right)=q_{i}$ be two distributions with compatible support. Then in the dual completed semifields issuing from $R_{\geq 0}$, the expression of the Rényi cross-entropy, entropy and divergence are:*

(41) $${\tilde{H}}_{r}\left(P_{X}\right)=I_{*}\left({\hat{M}}_{r}\left(P_{X},P_{X}\right)\right){\tilde{X}}_{r}\left(P_{X}∥Q_{X}\right)=I_{*}\left({\hat{M}}_{r}\left(P_{X},Q_{X}\right)\right){\tilde{D}}_{r}\left(P_{X}∥Q_{X}\right)=-I_{*}\left({\hat{M}}_{r}\left(P_{X},\frac{P_{X}}{Q_{X}}\right)\right)$$

Notice that when $F_{P_{X}}=\bar{n}$ and $F_{Q_{X}}=\bar{n}$ the definition of entropy based on the mean and in the semifield expressed are the same, whereas in the other cases there is an extension in the definition which is in agreement with several arbitrary choices made in the definition of the new means, e.g., $0\underset{\cdot }{⊗}\log0=0$ to comply with the convention extant for entropies. This supports our claim that entropies can be written and operated in semifields.

Since the equivalent probability function is a mean and the means can be expressed in a complete semifield, we have an expression of the former in the complete semifields of reals. Therefore, the expressions for the equivalent probability function and the information potential are:

(42) $$\begin{array}{cc} {\tilde{P}}_{r}\left(P_{X}\right)={\hat{M}}_{r}\left(P_{X},P_{X}\right)\; & {\tilde{V}}_{r}\left(P_{X}\right)={\hat{M}}_{r}{\left(P_{X},P_{X}\right)}^{r}. \end{array}$$

However, in the definition of the equivalente probability function, and the entropies, we have that $w_{i}=x_{i}$ which entails some of the cases in Figure 4 are not visited.

### 3.3. Discussion: A Conjecture on the Abundance of Semifields in AI, ML and CI Applications

We are now in a position to better sustain our main conjecture about the abundance of semifields in science domains that try to model intelligent behaviour:

**Conjecture** (Pervasiveness of positive semifields in modelling intelligence)**.** *Models and applications in Machine Intelligence—whether AI, ML or CI—operate with information, equivalent probability or proxies thereof, and those calculations are better conceptualized and successfully operated with the adequate dual pairs of positive semifields, especially entropy semifields.*

The argument for this conjecture is as follows:

1. First, the shifting in definition of the Rényi entropy by r=α−1 in [9] leads to a a straightforward relation (5) between the power means of the probability distribution and the shifted Rényi entropy. For a given probability function or measure $P_{X}$ the evolution of entropy with r∈[−∞,∞] resembles an information spectrum ${\tilde{H}}_{r}\left(P_{X}\right)$. In a procedure reminiscent of defining an inverse transform, we may consider an equivalent probability ${\tilde{P}}_{r}\left(P_{X}\right)=b^{-{\tilde{H}}_{r}\left(P_{X}\right)}$, which is the Hölder path of $P_{X}$, ${\tilde{P}}_{r}\left(P_{X}\right)=M_{r}\left(P_{X},P_{X}\right)$.
2. The function used by Rényi to define the generalized entropy, when shifted, is the composition of two functions: Hartley’s information function and the power function of order *r*, which are monotone and invertible in the extended non-negative reals [0,∞]. They are also bijections:
  - The power function is a bijection over of the extended non-negative reals, and
  - Hartley’s is a bijection between the extended reals and the extended non-negative reals.
3. But in Construction 1 both the power function and Hartley’s prove to be isomorphisms of positive semifields, which are semirings whose multiplicative structure is that of a group, while the additive structure lacks additive inverses. Positive semifields are all naturally ordered and the power function respects this order within the non-negative reals, being an order isomorphism for generic power *r*. Importantly, positive semifields come in dually-ordered pairs and the expressions mixing operations from both members in the pair are reminiscent of boolean algebras.
  (a) The power function $g\left(x\right)=x^{r}$ with r∈[−∞,∞]\{0} actually generates a whole family of semifields ${\left(R_{\geq 0}\right)}_{r}$ related to emphasizing smaller (with small *r*) or bigger values (with big *r*) in the non-negative reals $R_{\geq 0}$. Indeed, the traditional weighted means are explained by the Construction 2 as being power-deformed aritmetic means, also known as Kolmogorov-Nagumo means with the power function as generators. These, semirings come in dually-ordered pairs for orders *r*${\left(R_{\geq 0}\right)}_{r}$ and −r ${\left(R_{\geq 0}\right)}_{-r}$ whose orders are aligned or inverted with respect to that of $R_{\geq 0}$. Indeed, $R_{\geq 0}≅{\left(R_{\geq 0}\right)}_{1}$ (Corollaries 1 and 2).
  (b) However, Hartley’s function is a dual-order isomorphism, entailing that the new order in the extended reals is the opposite of that on the non-negative reals (Corollary 3). It actually mediates between the (extended) probability semifield $R_{\geq 0}$ and the semifield of informations, notated as a homage to Hartley as −H (Theorem 2).
4. Since the composition of the power mean and Hartley’s information function produces the function that Rényi used for defining his information measures, and this is a dual-order semifield isomorphism, we can see that entropies are actually operated in modified versions of Hartley’s semifields $H_{r}$ which come in pairs, as all completed positive semifields do (Theorem 3).
5. Many of the ${\left(R_{\geq 0}\right)}_{r}$ and $H_{r}$ semifields appear in domains that model intelligent behaviour. Among a list of applications we list the following:
  - In AI, maximizing utilities and minimizing costs is used by many applications and algorithms, e.g., heuristic search, to mimic “informed” behaviour ([5], Chapter 3), decision theory ([5], Chapter 16), uncertainty and probability modelling ([5], Chapters 13–15). In most applications ${\left(R_{\geq 0}\right)}_{1}$, for multiplicatively-aggregated costs and utilities, and $H_{1}$, for additively aggregated ones are being used. Note that both a semifield and its order-dual are needed to express mixed utility-cost expressions, as in electrical network analysis with resistances and conductances.
  - In ML, $R_{\geq 0}$ itself is used to model uncertainty as probabilities and H as log-probabilities. Sometimes the idempotent versions of these spaces $\lim_{r\to \pm \infty }{\left(R_{\geq 0}\right)}_{r}$, e.g., ${\bar{R}}_{\min,\times }$ and ${\bar{R}}_{\max,\times }$, and $\lim_{r\to \pm \infty }H_{r}$, e.g., ${\bar{R}}_{\min,+}$ and ${\bar{R}}_{\max,+}$, are used, e.g., for A* stack decoding to find best candidates [32]. The Viterbi algorithm operates in $H_{\infty }$, the semifield of max entropies, as required by the application of decoding Markov models ([25], and Section 3.2.1). Although many of the problems and solutions cited above for AI can also be considered as part of ML, a recent branch of ML is solely based upon the Rényi entropy with α=2, $H_{2}\left(P_{X}\right)={\tilde{H}}_{1}\left(P_{X}\right)$ [14]. Importantly, recall that every possible Hölder mean can be expressed as the arithmetic mean of a properly exponentiated kernel, whence the importance of this particular Rényi entropy would come.
  - In CI, the sub-semifield obtained by the restriction of the operations to {⊥,e,⊤} appears as a ternary sub-semifield distinct from the Boolean semifield, which in turn appears as a binary sub-semifield of every complete semifield by restricting the carrier set to {⊥,⊤}. As an important example, this ternary sub-semifield, as seen in Proposition 22 and Theorem 3 is pivotal in Spohn’s logical rank theory [33] that essentially leverages the isomorphism of semifields between $R_{\geq 0}$ and the ${\bar{R}}_{\min,+}\equiv H_{\infty }$ in logical applications.
    Finally—and also related to signal processing—mathematical morphology and morphological processing need to operate in the dual pair $\left({\bar{R}}_{\max,+},{\bar{R}}_{\min,+}\right)\equiv \left(H_{-\infty },H_{\infty }\right)$ for image processing applications [34].

In our opinion, these facts provide a substantive grounding for our conjecture.

### 3.4. Historical Notes: The Rise of Positive Semifields

Our first noted appearance of the addition operation of (20) in the context of information is by Barnard [35]. From a system of axioms—typical of the stage of development of Information Theory in those days—it was concluded that for the accumulation of probabilities p(x⊕y)=f(p(x),p(y)) the only possible functions are the (non-weighted) Kolmogorov-Nagumo (KN) means with $g\left(x\right)=x^{r}$, therefore the addition operation is $f\left(a,b\right)={\left(a^{q}+b^{q}\right)}^{\frac{1}{q}}$. A special note both prescribed that q≥0, since otherwise the function *f* is not strictly increasing, but cautioned against supposing that q=1 is the only possible value.

Rényi explicitly cites the work of Barnard when introducing his information measure [3], and his approach is well-known to have enlightened the relationship between the KN means and entropy. This introduces two considerations:

- From Barnard’s approach, it seems natural to use only α≥0 in the KN means, and this is what Rényi chose.
- It is more difficult to guess why he decided to place the “origin” of his entropies at Hartley’s (α=0) implying the harmonic average, instead of at Shannon’s α=1, related to the geometric average.
  He must have known about the theory of the means [12], but may have been doubtful about allowing negative values of his parameter, which he never discussed, e.g., in [10]. Since Hartley’s was already established as a measure of entropy, he may have felt that it was the “lowest” measure of entropy.

Notice there are no modern objections to using α<0 [36], and indeed the max-entropy α=−∞ is thoroughly used for specific applications. This led the authors of the present paper to define the shift in the order parameter that clarifies the relationship between the positive semifields in which they are defined vis-à-vis Pap’s g-calculus [9].

In what looks like yet another case of almost simultaneous discovery, the deformed averages of Barnard had been looked into as alternate models of algebra for the modelling of physical phenomena already by Grossman and Katz ([37], and references therein) and Burgin [38]. Due to our inability to gather more than the fundamental mathematics from Burgin’s paper, in Russian, we will review here the former work.

The non-Newtonian Calculus is an apt name by Grossman and Katz [37] to describe the behaviour of a systematic “deformation” of the standard algebra of the real numbers R. In this formalism, cast in the notation of this paper, an arithmetic is a complete ordered field A whose carrier set is a subset of R, and a generator α is a one-to-one function whose domain is R and whose range is a subset of it. Then the α-arithmetic is the complete ordered field obtained from the transformations:

- α-zero: $0_{\alpha }=\alpha^{-1}\left(0\right)$
- α-unit: $1_{\alpha }=\alpha^{-1}\left(1\right)$
- α-addition: $u{⊕}_{\alpha }v=\alpha \left(\alpha^{-1}\left(u\right)+\alpha^{-1}\left(v\right)\right)$
- α-subtraction: $u{⊖}_{\alpha }v=\alpha \left(\alpha^{-1}\left(u\right)-\alpha^{-1}\left(v\right)\right)$
- α-multiplication: $u{⊗}_{\alpha }v=\alpha \left(\alpha^{-1}\left(u\right)\times \alpha^{-1}\left(v\right)\right)$
- α-division: $u{⊘}_{\alpha }v=\alpha \left(\alpha^{-1}\left(u\right)/\alpha^{-1}\left(v\right)\right)$ for $v\neq 0_{\alpha }$
- α-order: $u\leq_{\alpha }v\Leftrightarrow \alpha^{-1}\left(u\right)\leq \alpha^{-1}\left(v\right)$

The properties of such α-arithmetics, and in particular the integral–differential calculus emanating from them, resemble formally the standard ones, since they operate in complete fields. The avowed intention of the authors is that “they may also be helpful in developing and understanding new systems of measurement that could yield simple physical laws ([37], p. 33).”

Indeed, this has been expanded upon and applied to alternate descriptions of physical phenomena by other authors [39]. The fundamental idea of this application is that the use of standard algebra to provide the “grounding” of integral-differential calculus for the description of physical phenomena is one symmetry (of nature) susceptible of “re-normalization”, e.g., for fractal description in a non-Newtonian calculus [40].

We can see two main differences the approach in [37] as compared to our own.

- First, the motivation for our construction, viz. the modelling of operations on information, is clearly more specialized thant Grossman and Katz’s, as purported in the above-mentioned quote.
- Second, and more importantly, in their framework, as in Barnard’s paper, α seems to be assumed monotone, and so the properties of antitone generators and their results (leading to the second half of the dual pair) in Pap’s g-calculus are downplayed.

However, regarding the second point, the order properties of dioids interact very tightly with their operations (see Section 2.2) and we believe this is the reason why Pap found it necessary to distinguish this case in his analysis on generator functions and their domains. As it happens, the resulting structure lacks an additive inverse which dovetails into the intuition that entropies and informations cannot be “(abstractly) subtracted”, hence we consider the construction in this paper more adequate for our purposes.

In any case it would be interesting to follow up on Grossman and Katz’s approach to particular “positive fields”. Since we have already proven that the geometries of their vector spaces are quite different in the, admittedly, extreme case of the idempotent (positive) semifields [15,30], it seems that some reinterpretation of this particular case should be undertaken in their formalism. We leave this for future work.

## 4. Conclusions

In the context of information measures, we have reviewed the notion of positive semifield—a positive semiring with a multiplicative group structure—distinct from that of the more usual fields with an additive group structure: in positive semirings there are no additive inverses, but there is a “natural order” compatible with addition and multiplication.

Through Pap’s *g*-calculus and Mesiar and Pap’s semifield Construction, we have related the Hölder means to the shifted Rényi measures of information ${\tilde{H}}_{r}\left(P_{X}\right)$ for pmf $P_{X}$, which appear as just the logarithm of the Kolmogorov-Nagumo means in different semifields obtained by ranging the *r* parameter in [−∞,∞]. As a fundamental example, we provide the rewriting of the Hölder means in ${\left(R_{\geq 0}\right)}_{r}$ and its dual, which provides the basis for the shifted Renyi entropy, cross-entropy and divergence.

Our avowed intention with this exploration was to provide a conjecture, from an information theoretic point of view, about the abundance of semifield valued quantities in a variety of machine learning and computational intelligence tasks. Namely, that such semifield-valued quantities are being used either directly as Rényi information measures—including Shannon’s—or indirectly as proxies of such. Experimental corroboration of this conjecture would seem to entail some form of constructive theory for intelligence.

## Figures and Tables

**Table 1:** Relation between the most usual weighted power means, Rényi entropies and shifted versions of them, from [9].

Mean Name	Mean $M_{r}\left(\vec{w},\vec{x}\right)$	Shifted Entropy ${\tilde{H}}_{r}\left(P_{X}\right)$	Entropy Name	α	*r*
Maximum	$\max_{i}x_{i}$	${\tilde{H}}_{\infty }=-\log\max_{i}p_{i}$	min-entropy	∞	∞
Arithmetic	$\sum_{i}w_{i}x_{i}$	${\tilde{H}}_{1}=-\log\sum_{i}p_{i}^{2}$	Rényi’s quadratic	2	1
Geometric	$\Pi_{i}x_{i}^{w_{i}}$	${\tilde{H}}_{0}=-\sum_{i}p_{i}\log p_{i}$	Shannon’s	1	0
Harmonic	${\left(\sum_{i}w_{i}\frac{1}{x_{i}}\right)}^{-1}$	${\tilde{H}}_{-1}=\log n$	Hartley’s	0	−1
Minimum	$\min_{i}x_{i}$	${\tilde{H}}_{-\infty }=-\log\min_{i}p_{i}$	max-entropy	−∞	−∞

## Abbreviations

The following abbreviations are used in this manuscript:

AI	Artificial intelligence
CI	Computational intelligence
KN	Kolmogorov–Nagumo
ML	Machine learning
